# Implementation of a Student-Teacher–Based Blended Curriculum for the Training of Medical Students for Nasopharyngeal Swab and Intramuscular Injection: Mixed Methods Pre-Post and Satisfaction Surveys

**DOI:** 10.2196/38870

**Published:** 2023-03-02

**Authors:** Julie Bieri, Carlotta Tuor, Mathieu Nendaz, Georges L Savoldelli, Katherine Blondon, Eduardo Schiffer, Ido Zamberg

**Affiliations:** 1 Faculty of Medicine, University of Geneva Geneva Switzerland; 2 Unit of Development and Research in Medical Education, Faculty of Medicine, University of Geneva Geneva Switzerland; 3 Division of General Internal Medicine, Department of Medicine, Geneva University Hospitals Geneva Switzerland; 4 Division of Anesthesiology, Department of Anesthesiology, Emergency Medicine, Clinical Pharmacology and Intensive Care, Geneva University Hospitals Geneva Switzerland; 5 School of Education Johns Hopkins University Baltimore, MD United States

**Keywords:** peer-learning, educator, education method, knowledge acquisition, training, student-teacher, COVID-19, nasal swab, vaccine, injection, skill assessment, skill development, vaccination, blended learning, blended education, hybrid education, medical education, pandemic, teaching, health care sector, medical student, hybrid learning, online learning, digital education, online education, online course, online class, simulation

## Abstract

**Background:**

The COVID-19 pandemic caused a major disruption in the health care sector with increased workload and the need for new staff to assist with screening and vaccination tasks. Within this context, teaching medical students to perform intramuscular injections and nasal swabs could help address workforce needs. Although several recent studies discuss medical students’ role and integration in clinical activities during the pandemic, knowledge gaps exist concerning their role and potential benefit in designing and leading teaching activities during this period.

**Objective:**

The aim of our study was to prospectively assess the impact in terms of confidence, cognitive knowledge, and perceived satisfaction of a student-teacher–designed educational activity consisting of nasopharyngeal swabs and intramuscular injections for the training of second-year medical students in the Faculty of Medicine, University of Geneva, Switzerland.

**Methods:**

This was a mixed methods pre-post surveys and satisfaction survey study. Activities were designed using evidence-based teaching methodologies based on the SMART (specific, measurable, achievable, realistic, and timely) criteria. All second-year medical students who did not participate in the activity’s old format were recruited unless they explicitly stated that they wanted to opt out. Pre-post activity surveys were designed to assess perception of confidence and cognitive knowledge. An additional survey was designed to assess satisfaction in the mentioned activities. Instructional design was blended with a presession e-learning activity and a 2-hour practice session with simulators.

**Results:**

Between December 13, 2021, and January 25, 2022, a total of 108 second-year medical students were recruited; 82 (75.9%) students participated in the preactivity survey and 73 (67.6%) in the postactivity survey. Students’ confidence in performing intramuscular injections and nasal swabs significantly increased on a 5-point Likert scale for both procedures—from 3.31 (SD 1.23) and 3.59 (SD 1.13) before the activity to 4.45 (SD 0.62) and 4.32 (SD 0.76) after the activity (*P*<.001), respectively. Perceptions of cognitive knowledge acquisition also significantly increased for both activities. For the nasopharyngeal swab, knowledge acquisition concerning indications increased from 2.7 (SD 1.24) to 4.15 (SD 0.83), and for the intramuscular injection, knowledge acquisition concerning indications increased from 2.64 (SD 1.1) to 4.34 (SD 0.65) (*P*<.001). Knowledge of contraindications for both activities increased from 2.43 (SD 1.1) to 3.71 (SD 1.12) and from 2.49 (SD 1.13) to 4.19 (SD 0.63), respectively (*P*<.001). High satisfaction rates were reported for both activities.

**Conclusions:**

Student-teacher–based blended activities for training novice medical students in commonly performed procedural skills seem effective for increasing their confidence and cognitive knowledge and should be further integrated within a medical school curriculum. Blended learning instructional design increases students’ satisfaction about clinical competency activities. Future research should elucidate the impact of student-teacher–designed and student-teacher–led educational activities.

## Introduction

The COVID-19 pandemic had a significant impact on health care delivery and caused important disruptions to medical education and training. These disruptions led to the realization of the major importance of clinical competency training at the pregraduate level, which is commonly based on in-person teaching activities and practical practice with peers on standardized and real patients. Access to all of these activities was restricted during most of the waves of the pandemic [1]. This forced medical schools and educators to reinvent teaching activities and use alternative and innovative ways for delivering education to ensure adequate training [2,3]. The methods typically used were videoconferences, e-learning modules, and other technology-based learning activities, which all proved to be efficient and beneficial during the pandemic [4-9].

However, clinical competencies require in-person teaching activities and practice, as mastering these skills is important for increasing students’ confidence to perform procedural skills and ensure patients’ safety [8]. In our institution, these activities are usually designed by senior physicians and led by student-teachers in the form of small group activities based on theoretical knowledge repetitions and low fidelity simulations. Student-teachers in our institution are medical students in their fourth to sixth year of medical school (in a 6-year curriculum).

The COVID-19 pandemic has put health systems worldwide under exceptional pressure and worsened an already existing shortage in medical staff [4,5,9-11] due to an overwhelming number of inpatient consultations, increased workload, as well as infected and quarantined health professionals [12,13]. Switzerland, for example, experienced a rapid deployment of national screening and vaccination programs, with more than 5700 vaccine doses administered daily [14] and more than 26,000 patients screened with nasal swabs [15]. This required additional workforce who could be rapidly trained to perform these procedures.

Within this context, teaching medical students to perform intramuscular injections and nasal swabs was a way to address this urgent need. However, as clinical medical educators were tied up in clinical activities related to the pandemic [4,9,16], the use of advanced medical students as student-teachers both for the design and as leaders of these activities might represent an interesting, viable, and valuable opportunity for the teaching of the abovementioned procedural skills and could eventually be extended to other similar activities within the medical school curriculum*.* Indeed, there is an increasing body of evidence claiming the pedagogical benefits of student-teacher–based activities in terms of improved critical thinking, learning autonomy, motivation, collaboration, and communication skills [17]. These benefits seem to apply not only to novice students but also to the student-teachers leading the activity [18-20].

Although several recent studies discuss medical students’ role and integration in clinical activities during the pandemic, knowledge gaps exist concerning their role and potential benefit in designing and leading teaching activities during this period [4,5,9]. Moreover, only a paucity of evidence exists concerning medical students’ perceptions and satisfaction from peer-designed and peer-led activities during the pandemic. The aim of our study was to prospectively assess the impact in terms of confidence, cognitive knowledge, and perceived satisfaction of a student-teacher–designed educational activity consisting of nasopharyngeal swabs and intramuscular injections for the training of second-year medical students (6-year medical school) in the Faculty of Medicine, University of Geneva, Switzerland. Our main hypotheses were that these activities would increase the perception of confidence and cognitive knowledge about these procedures among novice medical students and that students would be satisfied by the activity and its related instructional design.

## Methods

### Ethical Considerations

Ethics exemption was received by the Geneva Canton’s ethics committee as the project is outside the field of Human Research as described in the Federal Act on Research involving Human Beings. The exemption ID was REQ-2022-00453.

### Medical Curriculum and Local Health Care System

The medical school of the University of Geneva provides a 6-year medical curriculum. The first 3 years are considered preclinical with the main concentration on basic sciences, anatomy, physiology, and pathology. Teaching activities are for the most part conveyed in a problem-based learning instructional design. Nevertheless, the clinical competencies education starts from the second year with more than 80 educational activities and 5 formative assessments with standardized patients, with the goal of connecting scientific elements to clinical practice and preparing students for their clinical practice.

The medical school is affiliated with the Geneva University hospitals, which is the largest hospital in Switzerland and serves a regional population of more than 500,000 people. During the first 4 waves of the COVID-19 pandemic, the hospital handled more than 90% of the regional urgent and inpatient COVID cases. Medical students, as a regional policy, took active part in COVID-19–related care in different medical wards. Within this context, the mission of training novice medical students for nasopharyngeal swabs and intramuscular injections was given to the clinical competencies program team by the medical directors of the hospital and faculty with the purpose of increasing the potential workforce and alleviating pressure from the system.

### Study Population

This study included second-year medical students from the Faculty of Medicine in the University of Geneva, Switzerland. We excluded those who had already participated in the activity’s old instructional design and those who had explicitly stated they wanted to opt out of the study. The educational activities were mandatory, as it is a part of the regular medical school’s curriculum, and all eligible students were requested to participate. However, the participation in this study was on a voluntary basis and any student had the ability to opt out at any given moment. With regard to the transfer of knowledge to clinical practice, the participation in the screening and vaccination activities was on a voluntary basis, coordinated by the medical directorate of the hospital, and there were no sanctions imposed on the students.

### Student-Teachers

Fifteen medical students in their fourth, fifth, or sixth year of medical school are recruited each year to conduct 2 hours of practical training sessions for second-year and third-year medical students in the medical faculty of the University of Geneva. Student-teachers are recruited through a yearly call for applications. The selection process for student-teachers is done by the clinical competencies program’s faculty members and is based on academic achievements, teaching experience, and motivation. Each training session concerns the clinical competencies of a specific body system or a procedural skill. Before each session, including this study’s activities, student-teachers are trained by a senior specialist concerning the seminar’s specific theme.

### Role of Senior Experts

The clinical competencies program in our institution is run by a group of senior experts in different clinical domains. Each expert is responsible for all the training materials and activities concerning his/her domain of expertise (eg, Cardiology, Respiratory Medicine, Neurology). All the activities in our study were coordinated by IZ. Each expert will conduct a 2-hour yearly training session targeted at student-teachers to prepare them for their own teaching activities with novice students. Within the context of this study’s activity and due to time restrictions, 2 senior experts provided a 1-hour training for student-teachers and were present as backup during the activity. The activity’s material and support were designed and drafted by the student-teachers themselves with the supervision of the program’s coordinator IZ.

### Activity Design and Timing Considerations

Instructional design was blended with presession e-learning and video-based self-directed learning tasks followed by a 2-hour in-person practice session in small groups of 4-6 students using simulators. The time for the completion of presession tasks was estimated to be 60 minutes. During the practice session, 1 hour was dedicated to nasopharyngeal swab collection and 1 hour was focused on intramuscular injection. This study, including the training session and pre-post surveys, was conducted between December 13, 2021, and January 25, 2022. The learning objectives for the activity were drafted based on the SMART (specific, measurable, achievable, realistic, and timely) criteria [21], and all verbs mapped to Bloom’s taxonomy [22] for cognitive and psychomotor objectives (Table 1 and Table 2)*.*

**Table 1:** Learning objectives of the nasopharyngeal swab activity.

Number	Learning objectives
1	Cite the most frequent clinical situations indicating the performance of a nasopharyngeal swab.
2	List the main indications to perform a nasopharyngeal swab.
3	List the main contraindications to perform a nasopharyngeal swab.
4	Identify the anatomical landmarks on the dedicated model.
5	Cite the standardized sequence for performing a nasopharyngeal swab.
6	Prepare the personal protective equipment necessary to perform a nasopharyngeal swab.
7	Perform a nasopharyngeal swab on the mannequin or on a patient: install the patient, name the tube with the patient’s label, introduce the flexible swab into the nasal duct until it reaches the nasopharynx and make 3 rotations, close the tube, and send it to the laboratory.

**Table 2:** Learning objectives of the intramuscular injection activity.

Number	Learning objectives
1	Cite the two most frequent clinical situations indicating the performance of an intramuscular injection (drug administration, vaccine).
2	List the main indications to perform an intramuscular injection.
3	List the main contraindications to perform an intramuscular injection.
4	Name the 3 main complications of an intramuscular injection (local hematoma, allergic reaction, injection site infection).
5	Identify the different injection sites (deltoid muscle, large gluteal muscle, vast external muscle) on the mannequin or on a peer student.
6	Prepare the material to perform an intramuscular injection.
7	Perform an intramuscular injection: install the patient, maintain asepsis during the procedure, prick the deltoid/gluteus maximus muscle ensuring no reflux before injecting, apply a protective dressing, and monitor.

### Technology and Media Use

**Figure 1:**
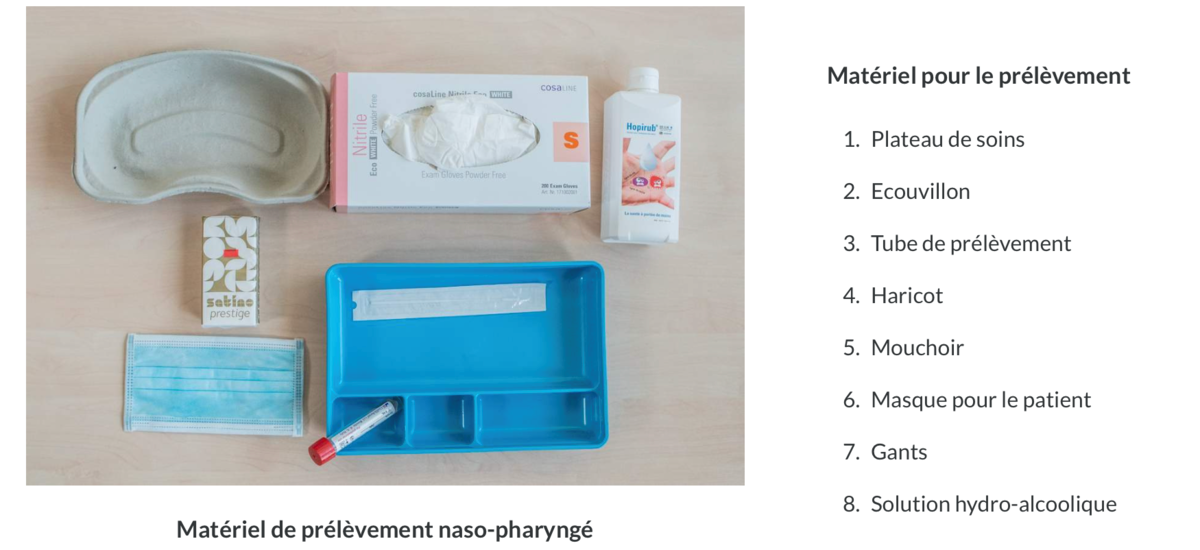
Cognitive visual aids for the swab technique in the e-learning module.

**Figure 2:**
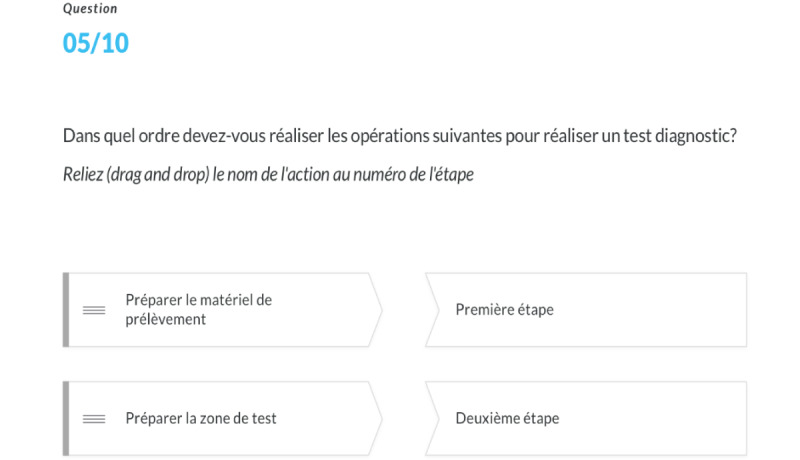
An example of the quiz evaluation for the clinical activity in the e-learning module.

The e-learning module for the nasal swab was created using Rise Articulate 360 (Articulate Global Inc) [23] and multiple-choice questions, text explanations, images, videos, and self-evaluation questions (Figures 1 and 2). 

### Practical Session/Activity

Random groups of 4-6 students were formed and led by student-teachers. The first 15 minutes were dedicated to discussing indications, contraindications, and hygiene measures for performing nasal swabs and intramuscular injections. This was followed by 10 minutes for demonstration of the procedural skill by the student-teacher, followed by dedicated time to perform the procedures on simulators under the supervision of the student-teacher.

### Activity Assessment

No formal assessment was done. Participation in the activity qualified as a passing grade.

### Pre-Post Activity Evaluation

To assess students’ perceptions of confidence in performing nasopharyngeal swabs and intramuscular injections as well as cognitive knowledge concerning indications and contraindications for both procedures, faculty members designed and validated presurveys and postsurveys. Answers to all surveys were based on a 5-point Likert scale (1=not at all, 2=rather not, 3=I don’t know, 4=rather yes, 5=perfectly) with the last question being an open-ended question for general comments. The preactivity survey was sent a few days before the activity and the postactivity survey was sent on the following day after the activity with several reminders to ensure an acceptable participation rate. An additional survey was created to assess students’ satisfaction in the designed activities.

### Postsession Satisfaction Survey

All students were given the opportunity to complete a 13-question web-based survey (Table 3) on their satisfaction based on a validated tool [24] to assess the perceived satisfaction and quality of the activity’s instructional design. The survey was created and disseminated using the LimeSurvey platform [25]. Answers to all questions were based on a 5-point Likert scale (1=I strongly disagree, 2=I disagree, 3=neither one nor the other, 4=I agree, 5=I completely agree) with the last question being an open-ended question for general comments. A score of ≥4 for each question was considered an acceptable rating of the activity’s quality.

**Table 3:** Satisfaction survey questions.

Number	Questions
1	I believe I acquired the learning objectives related to the nasopharyngeal swab and intramuscular injection.
2	The e-learning and simulation on the nasopharyngeal swab were effective and useful.
3	The e-learning on the nasopharyngeal swab was motivating and helped me to learn.
4	The e-learning combined with the simulation provided me with a variety of teaching methods allowing me to acquire the technical skills related to nasopharyngeal smear and intramuscular injection.
5	The practical training sessions on the nasopharyngeal smear and the intramuscular injection allowed me to acquire the knowledge and skills necessary for my immersion in the clinical environment.
6	These practical training sessions on the nasopharyngeal swab and the intramuscular injection are relevant during my learning curriculum.
7	I know how to use this simulation to remember the important elements of both technical procedures in case I have to perform them in the future.
8	I will know where to find the necessary references if I have any doubts about my skills in performing a nasopharyngeal swab or an intramuscular injection.
9	The student-teacher provided me with appropriate resources and references when needed.
10	The way the student-teacher taught the simulation was adapted to my way of learning.
11	The student-teacher’s responsibility is to give me constructive feedback on the technical gestures of the nasopharyngeal swab and the intramuscular injection.
12	The e-learning and simulation on the nasopharyngeal swab were effective and useful.
13	The e-learning on the nasopharyngeal swab was motivating and helped me to learn.

### Statistical Analysis

A mixed methods analysis was performed. Quantitative data were presented as mean (SD). We compared data between the 2 student groups by using the 2-sided *t* test for means. Stata version 16 (StataCorp LLC) was used for all statistical analyses [26]. A *P* value <.05 was used to indicate significance. Qualitative data were analyzed using a thematic analysis approach.

## Results

### Preactivity and Postactivity Survey Results

There were 192 eligible students. Out of them, 84 were excluded as they had participated in the activities in their old format. A total of 108 second-year medical students who met the inclusion criteria participated in both activities and were invited to answer the survey. Among them, 82 (75.9%) responded fully to the preactivity survey and 73 (67.6%) to the postactivity survey.

Students’ perception of knowing the indications for performing both procedures significantly increased from 2.7 (SD 1.24) to 4.15 (SD 0.83) for the nasopharyngeal swab (*P*<.001, Table 4) and from 2.64 (SD 1.1) to 4.34 (SD 0.65) for the intramuscular injection (*P*<.001, Table 5). We observed a similar increase in the contraindications, with the average score increasing from 2.43 (SD 1.1) to 3.71 (SD 1.12) and from 2.49 (SD 1.13) to 4.19 (SD 0.63), respectively. Novice students’ confidence in their ability to perform a nasal swab significantly increased from 3.59 (SD 1.13) before the activity to 4.32 (SD 0.76) after the activity (*P*<.001, Table 4, Figure S1 in Multimedia Appendix 1). Regarding intramuscular injection, confidence increased from 3.31 (SD 1.23) to 4.45 (SD 0.62) after the activity (*P*<.001, Table 5, Figure S2 in Multimedia Appendix 1).

**Table 4:** Survey questions and responses on nasopharyngeal swab.

Question	Preactivity survey (n=82), mean (SD)	Postactivity survey (n=73), mean (SD)	*P* value
I think I know all the indications for a nasopharyngeal swab.	2.7 (1.24)	4.15 (0.83)	<.001
I think I know all the contraindications for a nasopharyngeal swab.	2.43 (1.1)	3.71 (1.12)	<.001
I am confident in my ability to realize a nasopharyngeal swab.	3.59 (1.13)	4.32 (0.76)	<.001

**Table 5:** Survey questions and responses on intramuscular injection.

Question	Preactivity survey (n=85), mean (SD)	Postactivity survey (n=74), mean (SD)	*P* value
I think I know all the indications for an intramuscular injection.	2.64 (1.1)	4.34 (0.65)	<.001
I think I know all the contraindications for an intramuscular injection.	2.49 (1.13)	4.19 (0.63)	<.001
I am confident in my ability to realize an intramuscular injection.	3.31 (1.23)	4.45 (0.62)	<.001

### Satisfaction Survey Results

A total of 56 (51.2%) novice students responded to the satisfaction survey sent after the activity. Attainment of activity’s learning objectives was rated at 4.38 (SD 0.62) (Figure S3 in Multimedia Appendix 1) on a 5-point Likert scale. The ability of the blended learning design to help acquire competencies was rated at 4.11 (SD 0.71). The e-learning on the nasopharyngeal swab was considered effective and useful with an average of 4.3 (SD 0.69). Students perceived it as a motivating tool to learn, with a mean of 4.2 (SD 0.72) and found the practical activity relevant to their medical curriculum, with a rate of 4.54 (SD 0.76) on the 5-point Likert scale. Student’s ability to use the simulations to remember the important elements of both technical procedures in case of need to perform them in the future was rated at 4.27 (SD 0.67) and their ability to find the right information in case of doubt received a score of 4.29 (SD 0.82). Novice students considered the practical training sessions useful to acquire the knowledge and skills necessary for their immersion in the clinical environment with a mean of 4.34 (SD 0.67).

Regarding peer-to-peer teaching, providing constructive feedback on the procedural skills was considered a part of instructor’s roles, with a rate of 4.55 (SD 0.66) on a 5-point Likert scale. The student-teacher provided novice students appropriate resources and references when needed, with a mean of 3.75 (SD 0.98), and global appreciation of the student’s teaching was rated with a mean of 4.64 (SD 0.59). Novice medical students considered the instructional design of the activity to fit their study methods, with a rating of 4.57 (SD 0.68).

### Open-ended Answers

The topics raised in the open-ended answers could be summarized into 4 main themes (Table 6). Novice students seemed to have seized the relevance of the activity in the public health crisis and were happy to acquire competencies, which could help them participate in the collective effort. One student said, “Very relevant teaching in the context of the current pandemic. It was very interesting to receive this training to participate in the common effort in the vaccination and screening centers.” Moreover, students found the blended design useful to prepare for the practical activity. Further, students would have liked to have additional time to practice the procedures.

**Table 6:** Medical students’ open-ended answers (translated from French).

Theme	Illustrative quotes
Activity relevance	…*The module was really interesting and well explained, useful for my future career.* …*Very relevant teaching in the context of the current pandemic. It was very interesting to receive this training in order to participate in the common effort in the vaccination and screening centers.* …*Useful learning for the current health situation.* …*Useful and necessary session in times of pandemic.* …*Very good module. I work in a testing center and it was very helpful.*
Instructional design	…*E-learning is very useful to review the knowledge.* …*The practical part was a good summary of the e-learning main points, allowing a relevant synthesis of the information.*
Activity content	…*Regarding the content of the course’s written guide, more details would have been beneficial.*
Activity context	…*It should have been stated more specifically whether this was considered as training or as a formation to participate in the vaccination or screening campaign. The nasopharyngeal teaching was a bit quick (not much information in terms of indications and contraindications were recalled).* …*In general terms, it’s relevant to have integrated this in our curriculum, thanks!* …*The intramuscular injection was very well presented and conducted. …However, the nasopharyngeal smear was very quick, with few explanations. Considering the actual conditions, I understand the lack of time, but I would have liked more explanations and precisions on this invasive practice.* …*Very good and useful course. I would have appreciated a little more detail on indications and contraindications in some groups.* …*Take the time to explain how to prepare the vaccines (we only saw how to inject them) and how the SARS-CoV-2 antigen test works.* …*It was a very quick session, as well as all the technical procedure lessons we had. I don’t know if I’ll feel comfortable practicing it on my own.*

## Discussion

### Principal Findings

Our study aimed to examine the impact, in terms of confidence, cognitive knowledge, and satisfaction, of a student-teacher–designed and student-teacher–led activity to train second-year medical students at the University of Geneva, Switzerland, for nasopharyngeal swab collection and intramuscular injections during the COVID-19 pandemic. We provide several important insights in this study. First, the activity that was both designed and led by advanced students significantly increased the perception of confidence as well as cognitive knowledge among novice peers. Second, high scores in the satisfaction survey seem to indicate students’ acceptability for student-teacher–led activities for the teaching of basic clinical competencies. Third, the blended instructional design seemed to be effective for attaining learning objectives, increasing motivation, and providing callback references.

### Comparison to Prior Work

Recent studies have focused on the role and integration of medical students in clinical activities during the COVID-19 pandemic. However, there is a paucity of evidence concerning their potential role as teachers and instructional designers during and outside the context of the COVID-19 pandemic [4,5,9,27]. This role might be of interest during a pandemic period and could provide several benefits both to novice students (student-teachers) as well as the academic system [17]. In fact, many medical educators were overloaded with clinical activity, creating a shortage of workforce in academic settings. Advanced medical students with adequate supervision could help address this manpower gap in certain areas. In fact, in our study, the activities designed and led by students were effective in increasing novice peers’ confidence and cognitive knowledge for basic clinical competencies and were rated with high student satisfaction. Moreover, providing medical students with the role of a teacher and instructional designer might increase their motivation [18,19], promote continuous education, and develop the scholarship of teaching, as it will help them take control of their own curriculum and provide more value for the role of a teacher [8].

In addition, current body of evidence shows that student-teacher–led activities are beneficial not only to the learners by increasing their academic performance [28,29] but also to the student-teachers. In their systematic review, Yu et al [30] showed that peer teaching achieved similar short-term learners’ outcomes as the outcomes in activities run by the senior faculty. Moreover, systematic reviews showed a beneficial effect, both academically and professionally for student-teachers [29,30]. Similar results were reported by Benè and Bergus [29] in the context of problem-based learning and clinical skill activities, showing comparable performance in students trained by student-teachers versus those trained by faculty members. Their study [29] showed a positive impact of peer teaching on student-teachers themselves by enhancing their learning in relation to the content being taught. Our study reinforces these findings and provides more evidence to the beneficial effects of student-teachers. The integration of students as faculty support for clinical competencies teaching within and outside of crisis periods could be of value, and widespread implementation throughout the clinical medical curriculum should be considered.

### Potential Benefits of Blended Learning for Clinical Competencies Education

Blended learning is defined as a combination of traditional face-to-face learning and asynchronous or synchronous e-learning [31]. It was increasingly used as an instructional design during and outside the context of the pandemic [2] and was shown to be effective in terms of improving communication skills [32] and increasing students’ satisfaction [17,33,34] and confidence through the teaching activities [31,35,36]. Recent studies have shown the benefit of a blended design as well for the teaching of clinical skills [8,17,31,36]. Our study provides more evidence to support the usefulness of blended learning in this context. In fact, the use of the e-learning in our study in addition to the practice sessions was perceived as motivating and useful and provided a variety of teaching methods to stimulate learning. Indeed, a blended design for the teaching of clinical competencies can have several advantages. First, presession e-learning activities and providing interactive cognitive knowledge teaching can generate better preparation for the practice sessions, thus freeing up more time for the actual practice on simulators or with peers. Second, the designed material can be used as callback references and promote the use of high-quality and validated references by medical students in their curriculum. Finally, the designed material can be used by more advanced learners for training and callback and can potentially be translated to other disciplines such as nursing to enhance the training opportunities [17]. Therefore, the use of a blended learning design for clinical competencies teaching seems to be of value and should be further integrated within the medical school’s curriculum.

### Strengths and Limitations

Our study has several limitations. First, the sample size was small but did represent all the potential exposed students to the activity. In fact, all second-year medical students who did not already participate in the activities in their old format were eligible to participate in both activities. Second, the observational nature of the study could decrease the confidence in our results. Third, we did not have a control group of learners who were taught the same competencies with a different instructional design; however, as to the novelty of the activity’s design and the specific context of the pandemic, such control might not have been possible to establish. Future comparisons of students’ perceptions for the same activities run by senior experts versus those run by student-teachers would be of value to further assess the impact of student-teachers’ integration in teaching. Finally, we did not correlate our results of confidence, cognitive knowledge, and satisfaction to a measurement of performance. The measurement of performance as with the use of standardized scores in the form of Objective Structured Clinical Examinations could indeed be of value to attest the impact of student-teacher–led activities on clinically relevant outcomes and students’ preparedness for clinical practice. The latter will provide quantitative and standardized data, which could increase the confidence in our results. Due to the logistical constraints in the pandemic context, this was outside of the scope of our study; however, this will be the subject of our future work. The strengths of our study include the fact that the activities and related surveys were based on validated evidence-based tools and instructional design. In addition, the prospective recruitment of participants as well as the high participation rate in all the surveys represent important strengths of our work.

### Conclusions and Future Directions

The COVID-19 outbreak caused a major disruption within the health profession education and forced many institutions to reinvent teaching activities in a reality where the educational workforce was limited. Teaching of clinical competencies within this context represented an additional and unique challenge, as it required in-person teaching and introduction of new competencies to the curriculum. The use of student-teachers to lead and design such activities seemed to be effective to increase confidence and cognitive knowledge among medical students and resulted in high satisfaction ratings among learners. Blended learning design has the potential to increase learners’ satisfaction in clinical competency activities and provide more time for in-session practice. Designed teaching material could be introduced in postgraduate medical education and other medical disciplines. Further research should be performed to better understand the impact of student-teacher–led and designed activities on the quality of learners’ clinical competencies and their performance.

## Appendix

Multimedia Appendix 1 Survey results for nasopharyngeal swab and intramuscular injection activities.

## Abbreviations

SMART: specific, measurable, achievable, realistic, and timely

## Data Availability

The data sets generated during or analyzed during this study are available from the corresponding author on reasonable request.
