# Supplementary material for: Implementation of a Student-Teacher–Based Blended Curriculum for the Training of Medical Students for Nasopharyngeal Swab and Intramuscular Injection: Mixed Methods Pre-Post and Satisfaction Surveys
Source: JMIR Med Educ. 2023 Mar 2;9:e38870. doi: 10.2196/38870 (PMC10020911; doi:10.2196/38870)
Supplement: Multimedia Appendix 1 [file mededu_v9i1e38870_app1.docx]

**Figure S1.** Pre/postsurvey results for the nasopharyngeal swab activity.

| **Questions** | **Novice student’s answers** |
| --- | --- |
| I think I know all the **indications** for a nasopharyngeal swab | 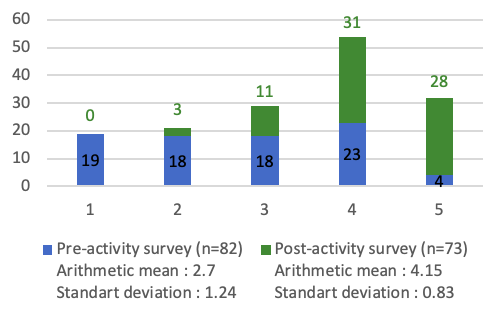 |
| I think I know all the **contraindications** for a nasopharyngeal swab | 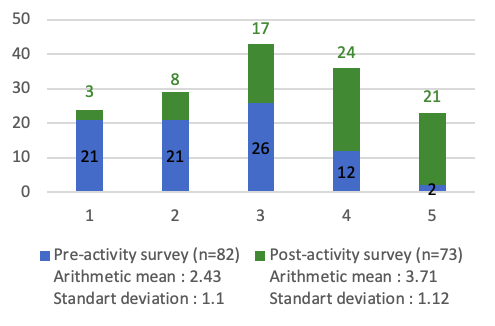 |
| I am **confident** in my ability to realize a nasopharyngeal swab | 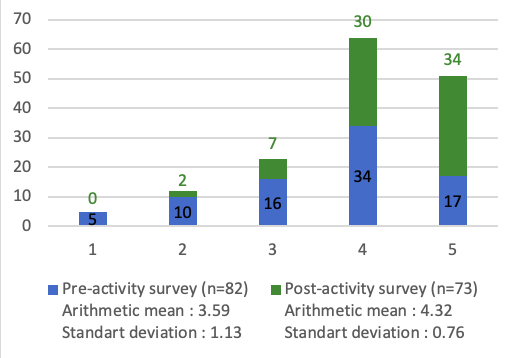 |

**Figure S2.** Pre/post survey results for the intramuscular injection activity.

| **Questions** | **Novice student’s answers** |
| --- | --- |
| I think I know all the **indications** for  an intramuscular injection | 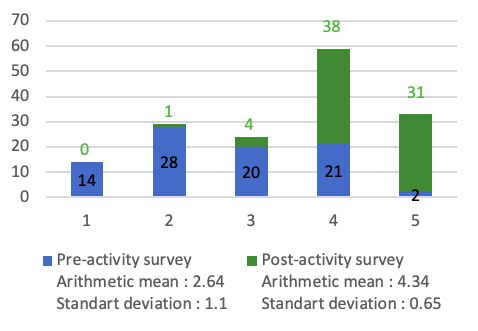 |
| I think I know all the **contraindications** for an intramuscular injection | 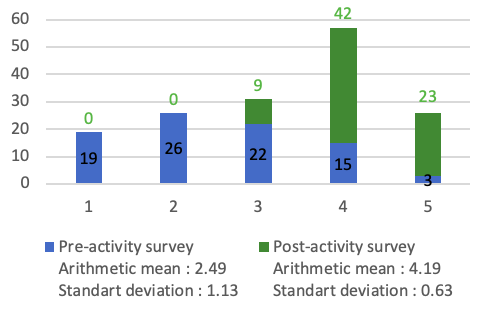 |
| I am **confident** in my ability to realize an intramuscular injection | 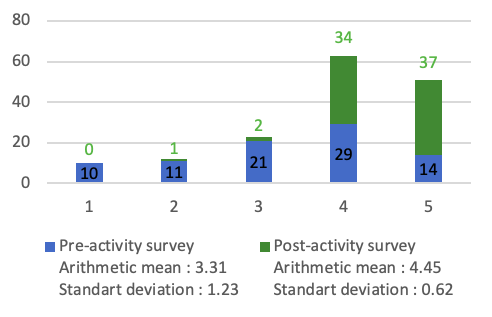 |

**Figure S3.** Satisfaction survey (translated from French)

| **Questions** | **Mean (SD)** |  |
| --- | --- | --- |
| I believe I acquired the learning objectives related to the nasopharyngeal swab and intramuscular injection. | 4.38 (0.62) | *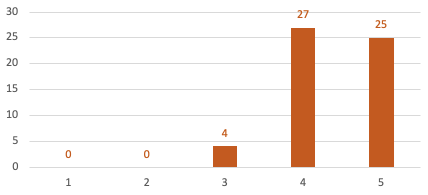* |
| The e-learning and simulation on the nasopharyngeal swab were effective and useful. | 4.3 (0.69) | *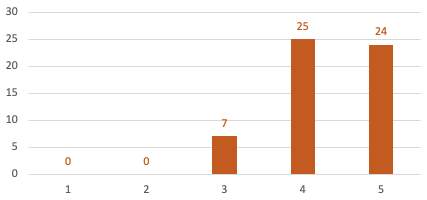* |
| The e-learning on the nasopharyngeal swab was motivating and helped me to learn. | 4.2 (0.72) | *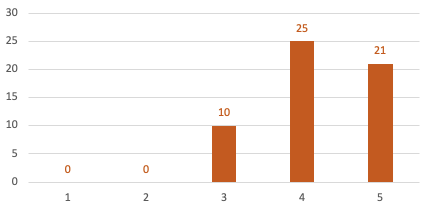* |
| The e-learning combined with the simulation provided me with a variety of teaching methods allowing me to acquire the technical skills related to nasopharyngeal smear and intramuscular injection. | 4.11 (0.71) | *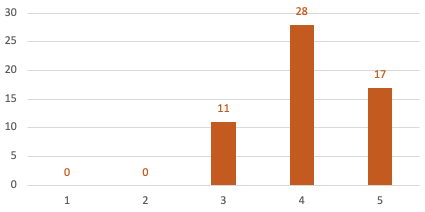* |
| The practical training sessions on the nasopharyngeal smear and the intramuscular injection allowed me to acquire the knowledge and skills necessary for my immersion in the clinical environment. | 4.34 (0.67) | *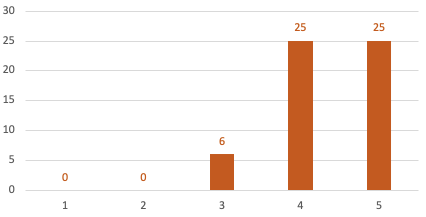* |
| These practical training sessions on the nasopharyngeal swab and the intramuscular injection are relevant during my learning curriculum. | 4.54 (0.76) | *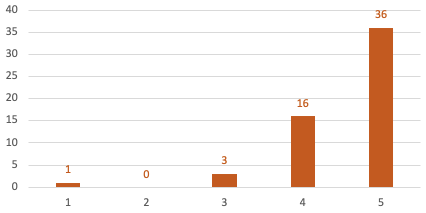* |
| I know how to use this simulation to remember the important elements of both technical procedures in case I have to perform them in the future. | 4.27 (0.67) | *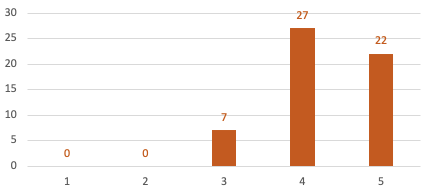* |
| I will know where to find the necessary references if I have any doubts about my skills in performing a nasopharyngeal swab or an intramuscular injection. | 4.29 (0.82) | *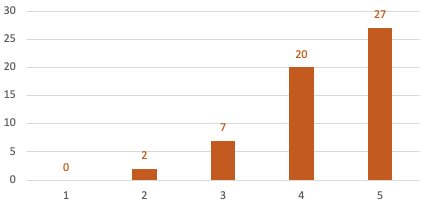* |
| The student-teacher provided me with appropriate resources and references when needed. | 3.75 (0.98) | *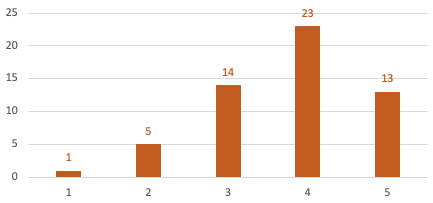* |
| The way the student-teacher taught the simulation was adapted to my way of learning. | 4.57 (0.68) | *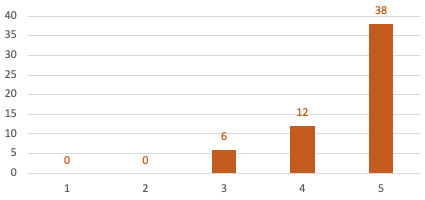* |
| The student-teacher’s responsibility is to give me constructive feedback on the technical gestures of the nasopharyngeal swab and the intramuscular injection. | 4.55 (0.66) | *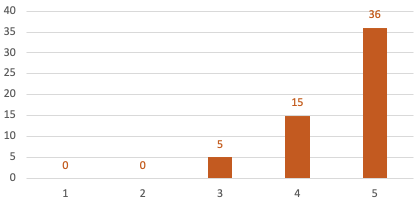* |
| I liked the way the student-teacher taught the simulation. | 4.64 (0.59) | *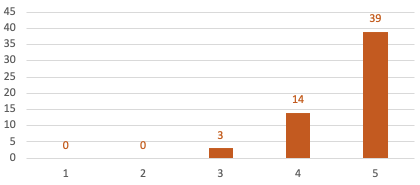* |
